# Whippets Causing Vitamin B12 Deficiency

**DOI:** 10.7759/cureus.23148

**Published:** 2022-03-14

**Authors:** Mani Maheshwari, Hemanthkumar Athiraman

**Affiliations:** 1 Hospital Medicine, Banner Health, Mesa, USA; 2 Hospital Medicine, Banner Health, Phoenix, USA

**Keywords:** vitamin b12 deficiency, subacute combined degeneration of the spinal cord, nitrous oxide myelopathy, nitrous oxide abuse, whippets

## Abstract

On the rise due to its effects of euphoria, recreational use of nitrous oxide can cause severe neurologic symptoms caused by marked and rapid onset of vitamin B12 deficiency. This is a case of a young 34-year-old male who presented with an inability to walk and confusion caused by vitamin B12 deficiency in the setting of recreational nitrous oxide use.

## Introduction

Recently, the recreational use of nitrous oxide has been increasing due to its euphoric effects [1]. Many obtain access to nitrous oxide as it is used in the food industry as a foaming agent and spray propellant in aerosol canisters, for example, those containing whipped cream [2]. Nitrous oxide can cause vitamin B12 deficiency, leading to glial cell dysfunction and subsequent demyelination of the central nervous system, and subacute combined degeneration of the spinal cord [3]. This is a case of a young 34-year-old male who presented with an inability to walk and confusion caused by vitamin B12 deficiency in the setting of recreational nitrous oxide use.

## Case presentation

A 34-year-old male with a history of alcohol use and dependence comes in by ambulance for generalized weakness. The patient is oriented to himself only, confused, and unsure why he is in the hospital. The patient’s father states he cleaned out 40,000 used nitrous oxide cartridges from the patient’s apartment, which he has used over the last seven to eight weeks (averaging use of >600 whippets/day). Vital signs are stable: blood pressure of 113/70 mmHg, heart rate of 95 bpm, respiratory rate of 18 breaths/minute, 98% pulse oximetry, the oral temperature of 37.4 degrees Celsius. Laboratory assessment shows mild macrocytic anemia (Table 1).

**Table 1:** Laboratory assessment on admission

Lab value	Result
Hemoglobin	12.5g/dL
MCV (mean corpuscular volume)	103 fL
Platelet count	127 K/uL
AST (aspartate aminotransferase)	60 U/L
Vitamin B12	215 pg/mL

Physical examination reveals norm muscle tone with 3/5 strength in upper extremities and 1/5 strength in lower extremities, oriented to only self, with nonsensical talk. As the patient can only intermittently follow simple commands, often falling asleep in the middle of the examination, we could not test memory or fund of knowledge.

**Figure 1:**
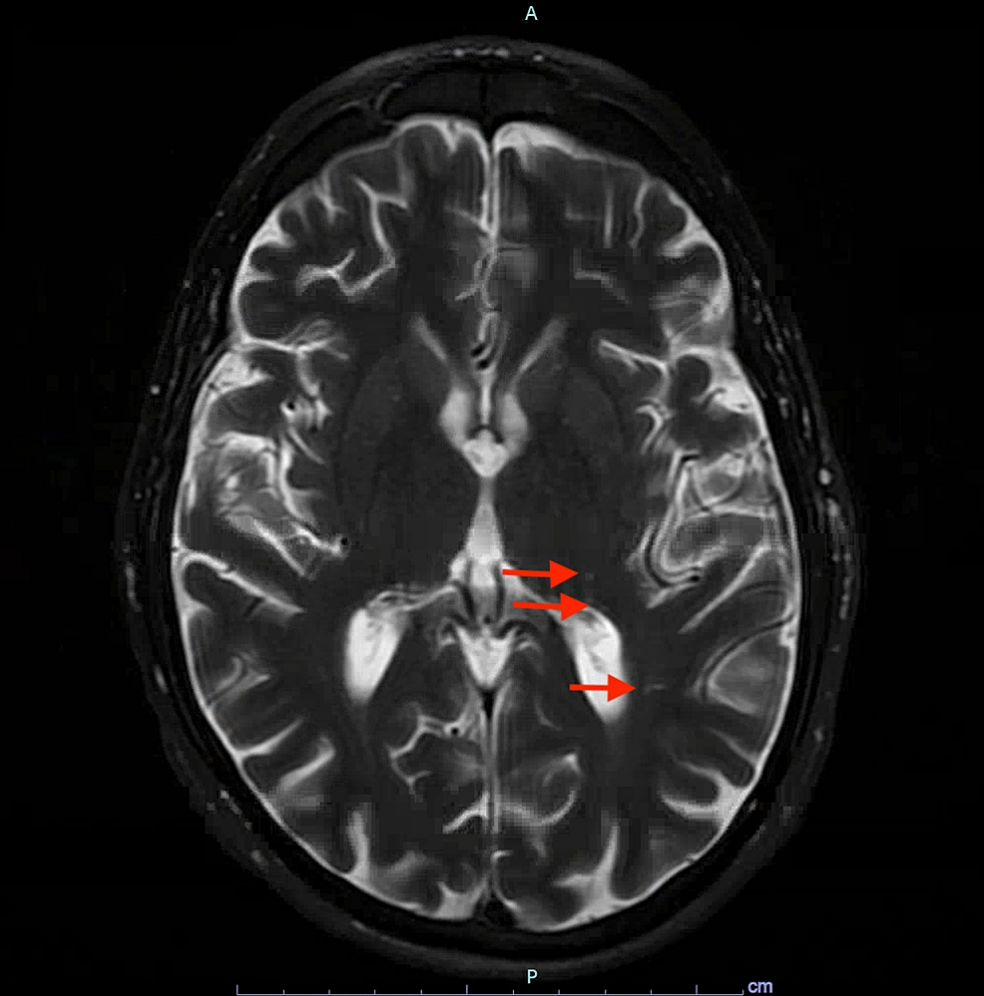
MRI of the brain Red arrows show the subtle scattered punctate foci of T2 prolongation in the left parietal lobe

The toxicology team is consulted and recommends starting vitamin B12 supplementation and ordering gas chromatography/mass ppectrometry. MRI of the brain shows findings consistent with toxic or metabolic leukoencephalopathy with hazy increased signal intensity in the bilateral corona radiata and bilateral posterior centrum semiovale, scattered punctate foci of T2 prolongation in the left parietal lobe, and moderate peripheral involution (Figure 1).

**Figure 2:**
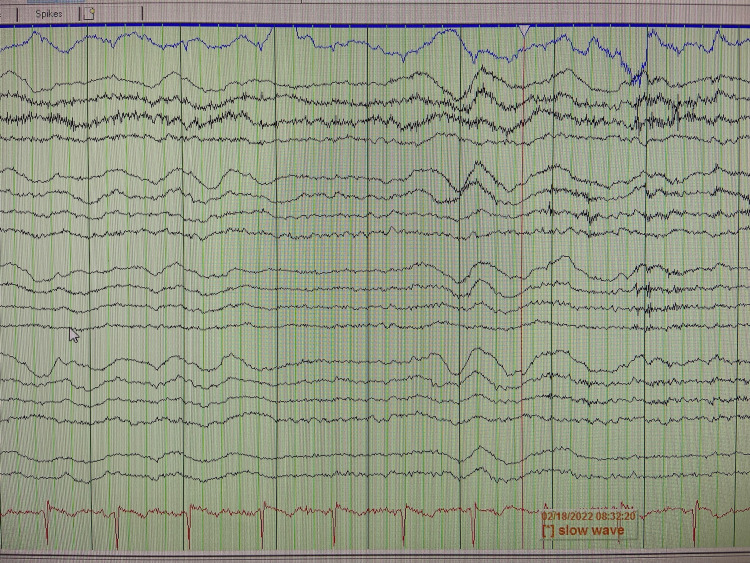
EEG (shows slow waves)

EEG shows mild diffuse cerebral dysfunction with intermittent, slow, generalized waves (Figure 2).

The next day, the patient seems to be more coherent but still has difficulty maintaining attention to one thought and is very tangential. Lower extremity weakness persists. Vitamin B12 supplementation continues, and the gas chromatography/mass spectrometry screen is unrevealing. On day six, the patient starts to improve neurologically, getting up with assistance and a walker for physical therapy evaluation. He improved, became oriented to the situation, and was discharged to acute inpatient rehabilitation for further therapy. 

## Discussion

Nitrous oxide toxicity results in vitamin B12 deficiency, which ultimately results in demyelination due to decreased normal myelin synthesis. A metabolite of cobalamin is a coenzyme in the reaction that catalyzes the conversion of methylmalonic CoA to succinyl-CoA - a substrate for the citric acid cycle. Nitrous oxide oxidizes the cobalt in vitamin B12 which irreversibly inactivates the cobalamin; hence, it is unable to function as a coenzyme. Methylmalonyl CoA then accumulates, entering into the lipid pathway, and incorporating abnormal fatty acids into neural lipids, which results in the decreased myelination of the lateral and posterior columns in the spinal cord and subacute combined degeneration. Patients with subacute combined degeneration of the spinal cord (dorsal and lateral columns) present with weakness, ataxia, and ascending paresthesia [4,5,6]. In a review of literature, it was found that patients who received general anesthesia with nitrous oxide had lower postoperative vitamin B12 levels compared to preoperative levels [7].

If normal vitamin B 12 levels are detected, it is important for providers to check methylmalonic acid and homocysteine levels-they will be elevated [6]. Early recognition and initiation of vitamin B12 supplementation results in the improvement of neurologic function. This occurred in this patient, and he was able to discharge to acute inpatient rehabilitation. Gas chromatography/mass spectrometry was unrevealing.

## Conclusions

It is vital for healthcare providers to recognize the connection between nitrous oxide use and vitamin B12 deficiency to timely supplement patients to relieve neurological deficits. Whether it is during an evaluation for vitamin B12 deficiency or nitrous oxide use, the provider should be aware of their connection.
